# Neutrophil-to-Lymphocyte and Platelet-to-Lymphocyte Ratio in Univentricular Patients From Birth to Follow-Up After Fontan—Predicting Lymphatic Abnormalities

**DOI:** 10.3389/fped.2021.740951

**Published:** 2021-12-08

**Authors:** Julia Moosmann, Christian Schroeder, Robert Cesnjevar, Kathrin Rottermann, Annika Weigelt, Sven Dittrich

**Affiliations:** ^1^Department of Pediatric Cardiology, University of Erlangen-Nürnberg, Erlangen, Germany; ^2^Department of Pediatric Cardiac Surgery, University of Erlangen-Nürnberg, Erlangen, Germany

**Keywords:** Fontan, MRI, univentricular heart defects, neutrophil/lymphocyte ratio, platelet lymphocyte ratio (PLR), lymphatic imaging, congenital heart disease

## Abstract

**Background:** Reliable laboratory parameters identifying complications after Fontan surgery including the lymphatic abnormalities and the development of protein-losing enteropathy (PLE) are rare. Neutrophil-to-lymphocyte ratio (NLR) and platelet-to-lymphocte ratio (PLR) are inflammatory markers and have been studied to predict outcome and prognosis in various diseases. The aim of this study was to investigate NLR and PLR from birth to follow-up after Fontan and evaluate their use as prognostic parameters for single ventricle patients regarding the development of lymphatic malformations during follow-up.

**Materials and Methods:** Sixty-six univentricular patients who underwent Fontan surgery and had 6-month follow-up magnetic resonance imaging (MRI) with T2 weighted lymphatic imaging after total cavopulmonary connection (TCPC) surgery were included in the study. NLR and PLR were determined at specific time points, from neonatal age to follow-up after Fontan operation and correlated to data from the MRI 6 months after Fontan.

**Results:** NLR and PLR increase significantly over time from the first surgery during infancy to the follow-up after Fontan (both *p* < 0.0001), with a significant increase after the Glenn surgery for both ratios (each *p* < 0.0001). Higher NLR (*p* = 0.002) and higher PLR (*p* = 0.004) correlated with higher-grade classification of lymphatic abnormalities in T2-weighted imaging 6 months after Fontan surgery and higher NLR correlated with higher transpulmonary gradient prior to Fontan surgery (*p* = 0.035) Both ratios showed a significant correlation to total protein at follow-up (NLR *p* = 0.0038; PLR<0.0001).

**Conclusion:** Increased NLR and PLR correlate with higher degree lymphatic malformations after TCPC and therefore might contribute as valuable additional biomarker during follow-up after TCPC. NLR and PLR are simple, inexpensive and easily available parameters to complement diagnostics after TCPC.

## Introduction

The Fontan procedure is a palliative surgical strategy for patients with functional single ventricle malformations. The Fontan circulation is characterized by a non-pulsatile pulmonary blood flow leading to increased central venous pressure and low cardiac output and a series of physiologic changes typical for the univentricular circulation (1–3). Treatment and care of patients with univentricular heart defects has undeniably made great progress in recent years. Despite the therapeutic achievements, Fontan patients continue to experience high complication rates after Fontan surgery including the development of the clinically challenging protein-losing enteropathy (PLE) and plastic bronchitis (4–7). The pathophysiological changes leading to Fontan failure and PLE are multifactorial and cannot yet be answered completely.

Changes in the lymphatic system increasingly emerged as a possible cause of Fontan failure and early complications including ascites, pleural effusions, PLE or plastic bronchitis within months after TCPC (8, 9). The increased central venous pressure in the Fontan circulation leads to an increase in lymph production and the development of lymphatic malformations. The lymphatic system can be visualized in T2-weighted imaging and has led to a classification of thoracic perfusion pattern by Biko et al. of four types describing the extent of lymphatic malformations, which are clinically associated with worse outcome (8, 10). Besides changes in the lymphatic system, PLE in Fontan patients is associated with inflammation and alterations in immune response (11). However, it is difficult to predict the occurrence Fontan failure and hemodynamic changes are often misleading here. Therefore, reliable laboratory parameters and biomarkers are needed in the follow-up care of Fontan patients to estimate the risk for Fontan complications.

Two ratios, which have emerged in recent years as markers of systemic inflammation are neutrophil-to-lymphocyte ratio (NLR) and platelet-to-lymphocyte ratio (PLR) (12).

NLR and PLR have been studied as prognostic markers in infectious diseases, postoperative complications, oncologic and cardiovascular diseases (e.g., myocardial infarction, admission with cardiac failure) to predict mortality and outcome (12–14). The preoperative NLR may also be used to estimate the outcome after Norwood or Glenn procedure (15, 16). Especially in the context of Fontan patients and protein loss, these seemed to be interesting markers, as they are not only inflammatory factors, but may also be influenced by changes in the lymphatic anatomy as seen in those patients.

The aim of this study is to illustrate the course of NLR and PLR over the period of Fontan palliation and to evaluate whether those ratios correlate with complications such as abnormalities in lymphatic perfusion pattern.

## Materials and Methods

### Study Population

**Figure 1:**
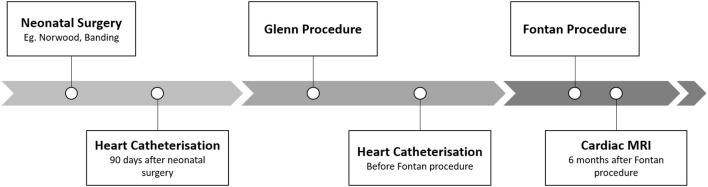
Anticipated pathway for univentricular patients undergoing the Fontan surgery.

A database search at the Department of Pediatric Cardiology at the Friedrich-Alexander-University Erlangen-Nürnberg revealed 76 Fontan patients, who underwent cardiac magnetic resonance imaging (MRI) after total cavopulmonary connection (TCPC) between 2007 and 2020, as part of our standardized in-house program for univentricular patients (Figure 1). The in-house protocol includes neonatal surgery (Aortopulmonary shunt, Norwood procedure, pulmonalarterial banding etc.), hemodynamic evaluation with heart catheterization 90 days after neonatal surgery (HC1), Glenn surgery, heart catheterisation for hemodynamic evaluation before the Fontan procedure (HC2), and TCPC (at the age of 3–4 years). Six months after TCPC a cardiac MRI is performed in our clinical routine to evaluate Fontan hemodynamics and T2-weighted imaging to illustrate lymphatic malformations. All patients, who did not undergo cardiac MRI and HC1 or HC2 have been excluded, with 66 univentricular patients remaining in the study. Patient demographics are displayed in Table 1. NLR and PLR values of Fontan patients were compared to the ratios of age matched patients from our laboratory database (excluding patients from NICU, PICU and patients with infections and increased CRP (≥5 mg/l) or PCT (≥0.5 ng/ml). The dataset consisted of 13,082 patients for NLR and 35,744 patients for PLR.

**Table 1:** Patient demographics, cardiovascular malformation, and patient history.

**Demographics**	**Number of patients**
**Gender**	
Female	22
Male	44
**Congenital heart malformation**	
Unbalanced atrioventricular canal (AVSD)	4
cc-TGA	5
Double inlet left ventricle (DILV)	10
Double outlet right ventricle (DORV)	6
Hypoplastic left heart syndrome (HLHS)	16
Pulmonary atresia intact ventricular septum (PA-IVS)	6
Single ventricle (SV)	3
Tricuspid atresia (TA)	16
Fenestration	16
**Systemic ventricle**	
Left ventricle (LV)	33
Right ventricle (RV)	30
Single ventricle type (SV)	3
**Patient history**	**age**
**Age at surgery**	
1^st^ surgery (d)	18.1 ± 25.9
Glenn procedure (m)	5.8 ± 5.5
Fontan procedure (y)	3.5 ±1.0
**Age at examinations**	
Heart catheterization before Glenn (d)	91.4 ± 29.8
Heart catheterization before Fontan (y)	2.9 ±1.1
Cardiac MRI after Fontan (y)	4.1 ±1.0

### MRI Protocol and MRI Analysis

MRI scans were performed as part of the routine follow-up for Fontan patients after TCPC. Scans were performed in supine position under general anesthesia with endotracheal intubation or laryngeal mask ventilation on a 1.5 Tesla MR scanner with high-performance gradients (Magnetom Aera, Siemens AG, Erlangen, Germany). The imaging protocol included balanced steady-state free precession cine sequences for functional and volumetric analysis of both ventricles. Phase-contrast MRI was acquired for the quantification of valvular regurgitation fractions. Contrast-enhanced MR angiography was conducted for three-dimensional depiction of the morphology of the ventricle, the pulmonary arteries, and the aortic arch. Scan parameters were as follows for all patients: slice thickness 8 mm, in-plane resolution 2.5 × 1.8 mm, time to echo 1.1 ms, time to repetition 42 ms, and flip angle 50 degree (17). T2-weighted lymphatic imaging was performed using a respiratory navigated and cardiac-gated three-dimensional turbo spin-echo sequence with the following parameters: 256 × 256; field of view, 300–450, repetition time/echo time 2,500/650; flip angle, 140 degrees; voxel size, 1.1 × 1.1 × 1.1 mm. The generated three-dimensional volume was formatted into a coronal maximal-intensity projection reconstruction of the source images.

Post processing image data analysis was performed using syngo.via (Siemens AG, Erlangen, Germany). Results of end-diastolic volume (EDV), end-systolic volume (ESV), stroke volume (SV), myocardial mass, and ejection fraction (EF) were documented. EDV and ESV were calculated by summing the volume of the ventricular blood pool in each section. EF was calculated based on EDV and ESV as [EF = (EDV– ESV)/EDV] × 100 %. T2-images were analyzed with the same software.

### Lymphatic Perfusion Pattern

Quantification of lymphatic perfusion pattern was performed according to the classification by Biko et al. by an experienced pediatric radiologist and pediatric interventional cardiologist. Classification includes type 1: little or no lymphatic abnormalities within the supraclavicular region and mediastinum, type 2: abnormal lymphatic perfusion pattern within the supraclavicular region without extension into the mediastinum, type 3: abnormal supraclavicular lymphatics with extension into the mediastinum, type 4: abnormal supraclavicular lymphatic channels with extension both into the mediastinum and into the lung parenchyma (10).

### Data Collection

We retrospectively evaluated demographic and clinical data and routine laboratory parameters collected as standard for every milestone of our protocol. Laboratory values are expressed over the entire surgical sequence from the neonate to TCPC and at MRI 6 months after Fontan surgery.

Clinical parameters were evaluated, including total hospital stay and the intensive care unit (ICU) stay at every surgery and the duration of drainages needed after the Fontan procedure (Table 2).

**Table 2:** Outcome parameters.

**Investigated parameters**	**Value**	***p*-value NLR**	***p*-value PLR**
**Hospital stay after 1**^**st**^ **surgery**			
Total (d)	21.19 ± 14.44	0.475	**0.046**
On ICU (d)	11.04 ± 9.57	0.760	0.542
**Hospital stay after Glenn**			
Total (d)	11.32 ± 11.52	0.970	0.409
On ICU (d)	3.41 ± 4.10	0.981	0.520
**Hospital stay after TCPC**			
Total (d)	27.21 ± 25.08	0.237	0.968
On ICU (d)	3.41 ± 4.34	0.790	0.446
**Early complications**			
Drainages after Fontan procedure (d)	22.35 ± 34.65	0.150	0.608
**Cardiac catheterization data**			
Glenn pressure (mmHg)	10.1 ± 2.8	0.777	0.290
Right pulmonary artery mean (mmHg)	9.3 ± 3.6	0.295	0.814
Left pulmonary artery mean (mmHg)	10.2 ± 3.2	0.315	0.313
End diastolic pressure (mmHg)	11 ± 5.6	0.134	0.087
Transpulmonary gradient (mmHg)	4.2 ± 2.3	**0.035**	0.098
**Data 6-month MRI**			
Ejection fraction (%)	46.35 ± 9.54	0.368	0.522
EDV (ml/m^2^)	98.71 ± 44.48	0.485	0.404
ESV (ml/m^2^)	56.28 ± 38.67	0.687	0.518
SV (ml/m^2^)	41.87 ± 11.62	0.262	0.144
**Classification of thoracic lymphatic malformations**		**0.002**	**0.004**
Type 1	15		
Type 2	20		
Type 3	21		
Type 4	10		

*TCPC, total cavopulmonary connection; RACHS-1, Risk Adjustment for Congenital Heart Surgery-1; ICU, intensive care unit. Values are expressed as mean ± SD. Statistically significant results were highlighted in bold*.

We retrospectively evaluated if early complications according to the definition of Gosh et al. occurred (18). Early complication include one of the following events within 6 months of TCPC: (1) death, (2) Fontan-takedown, (3) ECMO, (4) chest tube drainage >14 days, (5) cardiac catheterization, (6) readmission, or (7) listing for heart transplant.

The null hypothesis for this study was that there is no association of NLR and PLR at predicting lymphatic malformation or correlating with early complication after the Fontan surgery.

### Analysis of NLR and PLR

NLR and PLR were calculated from the absolute number of neutrophils, platelets and lymphocytes. Calculated by the number of neutrophils or platelets divided by the number of lymphocytes. Leucocyte subsets and total blood counts were measured on a Sysmex XE 2100 hematological analyzer (Sysmex Europe, Norderstedt, Germany), in accordance with standard operating procedures. All measurements have been performed in the pediatric laboratory of the Department of Pediatrics and Adolescent Medicine, University Hospital Erlangen, Germany. Both machine-counted and microscopic leukocyte subtypes were used for analyses. If both data sets were available, the microscopic findings were used. The time points of NLR and PLR analysis were chosen at scheduled routine investigations prior (date of admission) to operations or interventions in order to exclude acute effects of inflammation or complications associated with the surgical or catheter intervention. All patients were healthy and showed no clinical sign of infection prior to surgery, cardiac catheterisation or cardiac MRI.

### Statistical Analysis

Changes over time for NLR and PLR were calculated by variance analysis for repeated measurements. *T*-tests and a variance analysis for paired measurements were performed to test for influences between ventricular morphology and NLR/PLR. The quantitative influence variables were tested by correlation coefficients and multiple regression analysis. Correlations were calculated with Pearson and Spearman when appropriate. All statistical analyses were conducted using SAS, release 9.4 (SAS institute Inc., Cary. NC, USA). The results of the statistical tests have been considered as statistically significant if the *p* < 0.05.

### Ethical Statement

This study was conducted in accordance with the Declaration of Helsinki. The retrospective evaluation was approved by our local ethics committee at the Friedrich-Alexander University of Erlangen-Nürnberg. Patient consent to participate was not needed.

## Results

**Figure 2:**
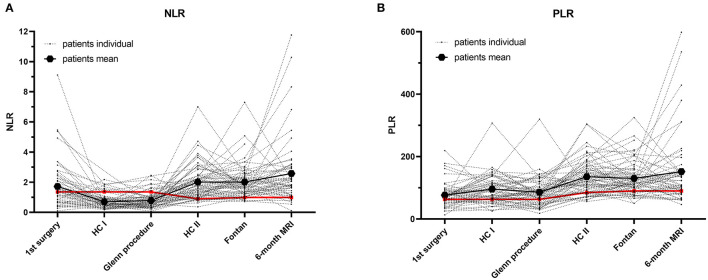
Individual courses of neutrophil-to-lymphocyte ratio (NLR) and platelet-to-lymphocyte ratio (PLR) of univentricular patients undergoing Fontan pathway. **(A)** NLR. **(B)** PLR. Mean of control group is illustrated in red. Mean values of all Fontan patients are given in thick black.

**Figure 3:**
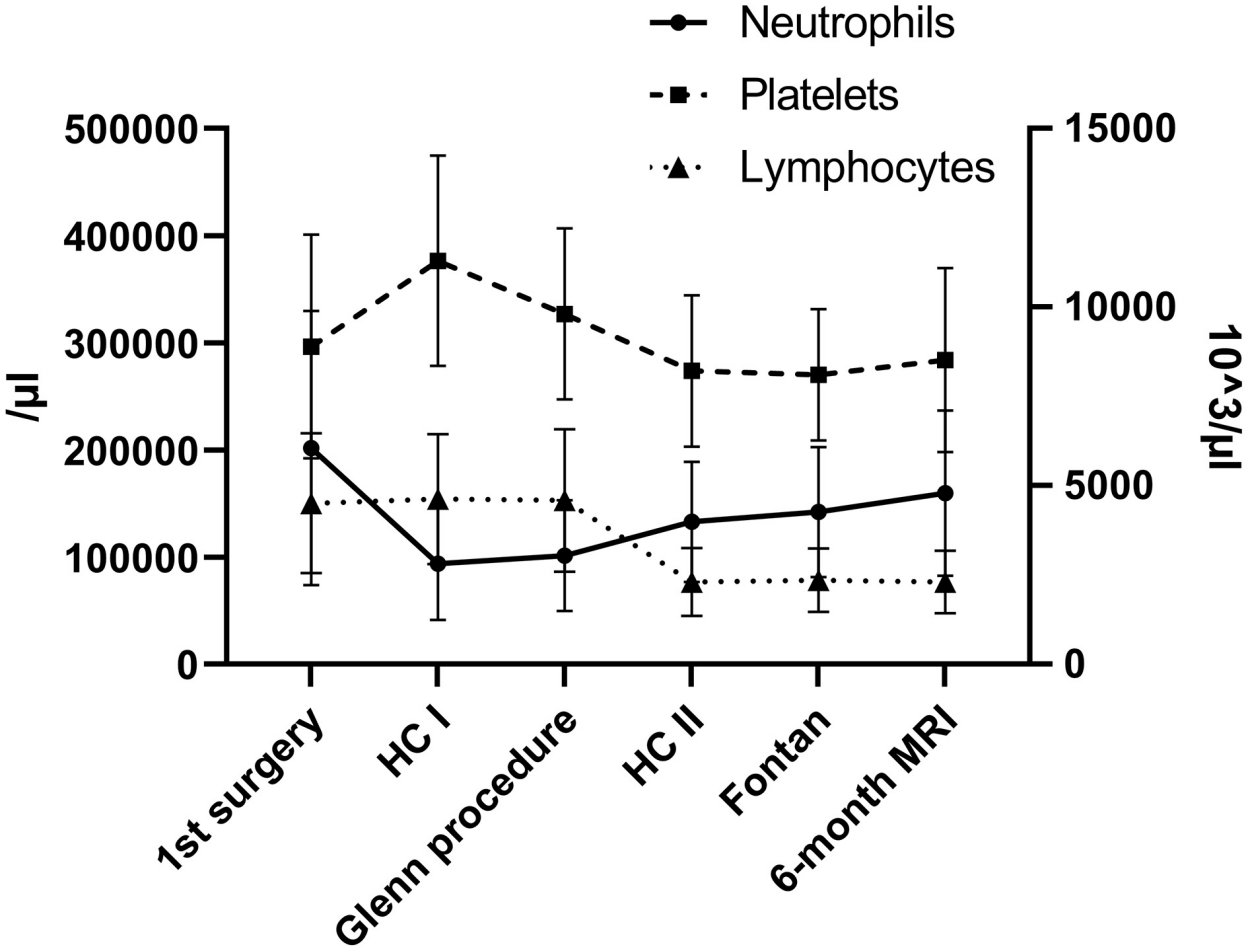
Neutrophils, platelets and lymphocytes during the course of Fontan palliation.

We analyzed 66 Fontan patients who successfully underwent our in-house protocol for univentricular malformations from birth to TCPC and 6-month MRI during follow-up. Underlying cardiac malformations and patient demographics at different time points are illustrated in Table 1. Our routine interventions were performed according to our protocol: 90-day heart catheterisation after the first surgery, cardiac catheterisation before Fontan and 6-month cardiac MRI after TCPC (Figure 1 and Table 1). Our analysis illustrated the progression of two ratios, NLR and PLR, over the first 4–5 years of univentricular patients at those specific time points. We observed a significant increase of NLR (*p* < 0.0001) and PLR (*p* < 0.0001) over time from birth to follow-up (Figures 2A,B). NLR is increased in the neonatal age and first months of life, compared to the older ages. Values for NLR and PLR show a significant increase after the Glenn procedure (*p* < 0.0001 and *p* < 0.0001, respectively). After the Glenn procedure, both values were significantly higher compared to the age matched controls (Figures 2A,B). Looking at the individual courses, it becomes apparent that there is an increase after the Glenn operation in all patients, but ~15% of patients show an exponential increase of NLR>3 and 12% an increase of PLR >200. Four patients showed both NLR>3 and PLR>200 after Glenn surgery. In 7/10 patients NLR normalized before Fontan surgery and 4/8 patients PLR normalized before Fontan surgery. After Fontan surgery a significant increase of PLE>200 (9/66 patients) and NLR >3 (11/66 patients) was observed, including 5 individuals with both parameters elevated (Figures 2A,B). The individual course of neutrophils, lymphocytes and platelets are illustrated in Figure 3. The morphology of the systemic ventricle did not show any correlation on NLR (*p* = 0.267) or PLR (*p* = 0.985). There was no correlation between NLR or PLR concerning time on ICU. We found a correlation between longer total hospital stay after Fontan surgery and higher NLR (NLR: *p* = 0.025 but not PLR: *p* = 0.372; Table 2). After the Fontan surgery we did not observe a correlation between duration of drainages and PLR (*p* = 0.150) or NLR (*p* = 0.608).

**Figure 4:**
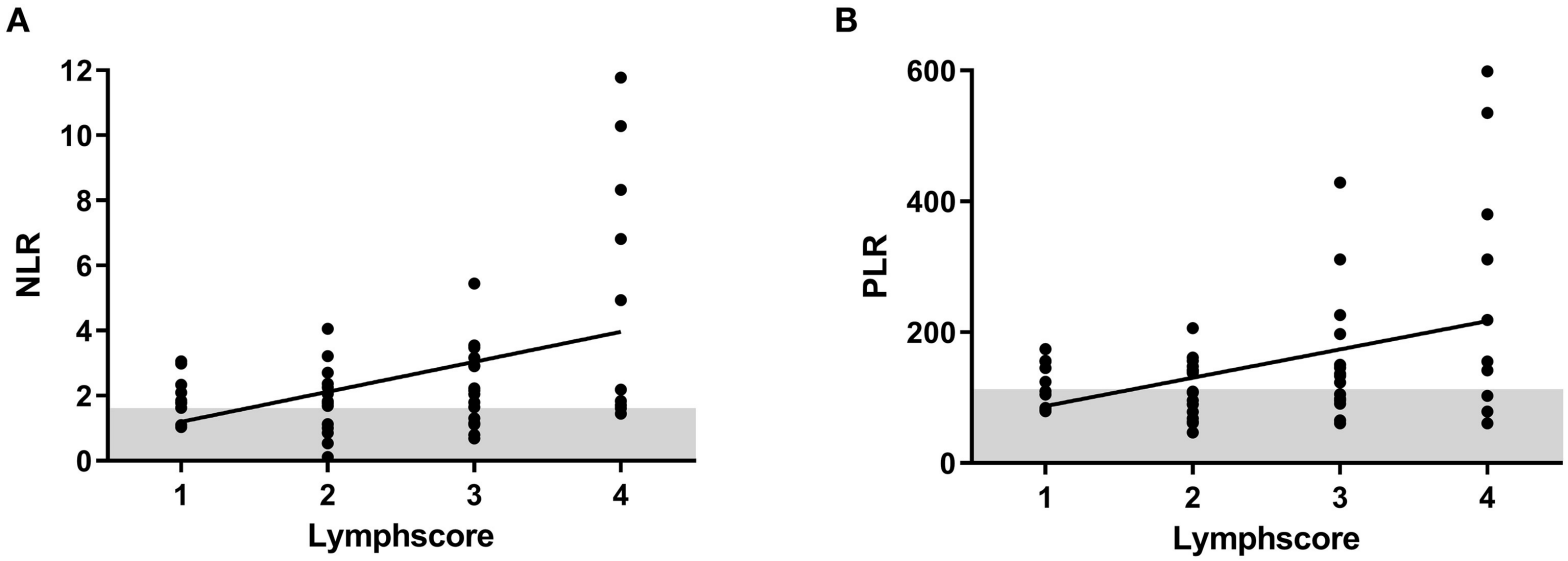
Correlation of neutrophil-to-lymphocyte ratio (NLR) and platelet-to-lymphocyte ratio (PLR) to classification of lymphatic abnormalities from T2-weighted imaging data at 6-month magnetic resonance imaging (MRI). **(A)** NLR. **(B)** PLR.

**Figure 5:**
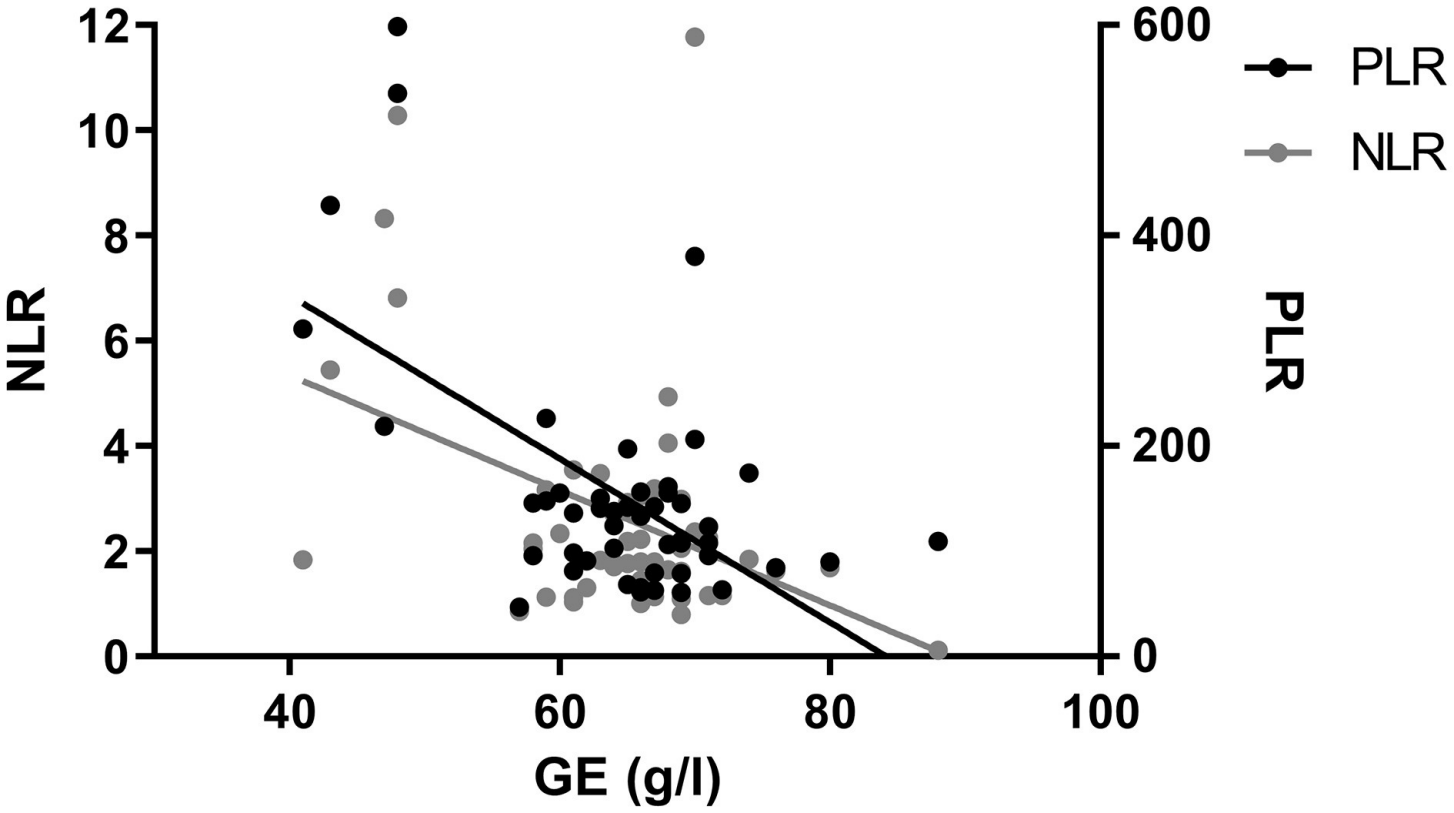
Correlation of neutrophil-to-lymphocyte ratio (NLR) and platelet-to-lymphocyte ratio (PLR) with total protein (TP) concentration. NLR and PLR correlate significantly with total protein (*p* < 0.0001 and *p* < 0.0001) at 6-month MRI.

Results of the 6-month MRI were correlated to NLR and PLR at that time. NLR as well as PLR correlated significantly to lymph score of Biko et al. (NLR: *p* = 0.0019; PLR: *p* = 0.0038; Figures 4A,B). Patients with type 3 or 4 thoracic lymphatic malformation showed NLR values above the 97.5 percentile (type 3: 2.6 ± 2.2; type 4: 4.4 ± 3.9) and compared to patients with lymphatic classification of 1 or 2 who showed normal NLR values (type 1: 2.2 ± 0.7 and type 2: 1.9 ± 1.1). No other significant correlations to results of the MRI could be found (Table 2). We found a significant correlation between total protein and NLR and PLR at the 6-month MRI (NLR: *p* < 0.0038; PLR <0.0001; Figure 5). No significant correlation has been found between NLR/PLR and total protein at HKU2 after Glenn (NLR: *p* = 0.167; PLR = 0.348 and at TCPC (NLR: *p* = 0.174; *p* = 0.182).

The cardiac catheterization data pre-Fontan was analyzed (Table 2). A significant correlation could be found between transpulmonary gradient (TPG) and the degree of lymphatic malformation in 6-month MRI (*p* = 0.009) as well as a significant correlation between TPG and NLR at the time of MRI. No significant correlation was found between TPG and NLR at the time of cardiac catheterization and before TCPC. No significant correlation was found for PLR at any time point.

*N* = 44 patients developed early complication. *N* = 2 patients required postoperative ECMO therapy. *N* = 31 patients presented with chest drain >14 days. *N* = 15 patients required additional cardiac catheterization and *n* = 11 patients required additional hospitalization within the first 6 months after TCPC. Patients who presented with higher grade lymphatic malformations type 3 or 4 (*n* = 31) *n* = 24 (77%) had an early complication and *n* = 8 (26%) at least two early complications. Of the lower grade lymphatic malformations type 1 and 2 (*n* = 35) *n* = 20 (57%) had an early complication with *n* = 3 patients (9%) showing two or more early complications.

## Discussion

In this study, we analyzed the course of NLR and PLR from first surgery to cardiac MRI 6 months after Fontan surgery to evaluate whether NLR and PLR would suit as potential and easily accessible laboratory markers for Fontan patients at risk for the development of lymphatic malformations. We identified a significant increase of both ratios during the palliation process with a significant rise after the upper cavopulmonary connection and TCPC including a correlation to the degree of lymphatic perfusion pattern in the 6-month MRI.

The interplay between lymphatic malformation and hemodynamic changes in the Fontan circulation are gaining increasing scientific interest in recent years. Biko et al. suggested a classification for neck and thoracic lymphatic abnormalities from T2-weighted MRI data (10). Higher-grade abnormalities in lymphatic perfusion pattern have been associated with early complications after Fontan surgery and Fontan failure (8, 10). Further, it was demonstrated that abnormalities of thoracic lymphatic perfusion pattern already occur after upper cavo-pulmonary connection (Glenn surgery) despite they often do not manifest clinically (18). The lymphatic system including lymphatic vasculature and lymph fluid represents a major player regulating immune function and tissue fluid balance. Which is of particular interest in Fontan patients, who show immunologic alterations including lymphopenia and changes in T cells and T cell subsets (11). Lymphopenia in Fontan patients has been associated with portal hypertension and PLE and an association to lymphatic malformations has been postulated (19, 20). However, the interplay between lymphatic abnormalities and lymphopenia in Fontan patients is not fully understood.

We now identified a correlation between the severity of thoracic lymphatic classification (Type 1–4) and higher levels of NLR and PLR at 6-month MRI. We suggest that the changes in lymphatic perfusion pattern lead to cellular changes especially the observed decrease in the absolute number of lymphocytes. The increase of neutrophils in Fontan patients might be another underlying contributor to Fontan failure and chronic inflammation as seen in other chronic diseases (21). Further, we observed a significant increase in NLR and PLR after the Glenn procedure, with a specifically marked increase for selected patients. We hypothesize that this might also correlate to hemodynamic and lymphatic changes after upper cavopulmonary connection, as described earlier (18). We do not have T2-weighted imaging data to support this hypothesis and this needs to be addressed in a further study, however we have been able to show that higher TPG prior to Fontan is associated with higher NLR and also higher-grade lymphatic malformations. The pathogenesis of the lymphatic changes in Fontan patients is not fully understood, however, increased central venous pressure is discussed to overwhelm the lymphatic circulation causing increased lymphangiogenesis and lymphangiectasia. Further, lympangiogenesis occurs during inflammation as Vascular Endothelial Growth Factor C (VEGF-C) a major promotor of lymphatic growth, is produced not only by lymphatic endothelial cells (LECs) but inflammatory cells. In addition, neutrophils can release pro- and anti-inflammatory cytokines through the myeloperoxidase pathway leading to endothelial dysfunction, increased microvascular permeability, tissue damage and have been identified to produce VEGF-A and D coordinating lymphangiogenesis (17, 22). Therefore, an increase in neutrophils as we have observed in Fontan patients (together with decreasing lymphocytes contribute to a higher NLR) might be a good marker for lymphatic changes. The importance of neutrophils in Fontan patients is also demonstrated by the study of Geanacopouloshere et al. where pulmonary casts of patients with Fontan circulation and plastic bronchitis were studied. Contrary to previous considerations, these were predominantly neutrophilic, proteinaceous casts with a concomitant elevated neutrophil cell count in the broncho-alveolar lavage (BAL), suggesting that inflammation might play a role in cast development and potentially Fontan complications such as PLE (23). For PLE this has not been investigated in detail yet. Total protein is used as a laboratory routine parameter especially for monitoring for PLE in Fontan patients. Both ratios showed a good correlation with total protein levels after TCPC, but interestingly not after Glenn. After anastomosis of the inferior vena cava the abdominal venous pressure increase leading to changes in hepatic flow characteristics unique to the Fontan circulation (24). This might affect liver synthesis as seen in lower total protein and is not observed after Glenn surgery. Since the follow-up time after Fontan is only 6 months, a comprehensive statement about the occurrence of PLE and correlation to the two ratios is in our opinion not yet possible in this patient group.

However, the timely correlation of lymphatic dysfunction and NLR and PLR needs further investigation including T2-weighted imaging after the upper cavopulmonary connection and during a longer follow-up period. In particular, the increase after Glenn surgery seems to be transient in some patients and return to normal before Fontan surgery. Whereas, especially after Fontan, there is an excessive increase in NLR and PLR. Based on our data, we cannot conclusively judge whether this is a longer-lasting effect or also transient. Considering, that the lymphatic classification focuses solely on neck and thoracic malformations, there might be an additional bias regarding patients at risk for PLE, as intestinal and mesenteric lymphatic malformations are not classified yet. High PLR has also been shown to be associated with poor prognosis in various diseases e.g., several cancers and coronary artery disease. However, the Fontan circulation differs significantly form the biventricular circulation with a well-documented increased risk of thrombosis, despite decreasing platelet counts over time (25). Although platelets decrease over the course of palliation, the pronounced decrease in lymphocytes leads to an increase in PLR. Based on our data, we cannot say whether an increased PLR is associated with an increased risk of thrombosis.

Clinically, we suggest calculating NLR and PLR for univentricular patients during their course to TCPC and during follow-up. Patients who have a higher NLR and PLR need to be critically evaluated for other signs of Fontan complications including lymphatic malformations and PLE and may require closer monitoring.

### Limitations

We analyzed NLR and PLR ratios at scheduled routine time points in the course of staged treatment of univentricular hearts, however we cannot comment on the potential influence of perioperative parameters, specific complications or modifiable risk factors to prevent lymphatic malformations and thus higher NLR and PLR.

## Conclusion

NLR and PLR correlate with the degree of lymphatic malformations in T2-weighted imaging and might contribute as new laboratory markers for Fontan complications. Both ratios show a significant increase after Glenn surgery, suggesting a correlation to the altered hemodynamics and possibly lymphatic circulation. NLR and PLR are simple, inexpensive and easily available parameters to complement Fontan diagnostics during the steps of palliation and during follow-up.

## Data Availability Statement

The raw data supporting the conclusions of this article will be made available by the authors, without undue reservation.

## Ethics Statement

The studies involving human participants were reviewed and approved by Friedrich-Alexander University of Erlangen-Nürnberg. Written informed consent from the participants' legal guardian/next of kin was not required to participate in this study in accordance with the national legislation and the institutional requirements.

## Author Contributions

JM and SD conceived and designed the analysis. JM and CS acquired the necessary data. JM, SD, and CS drafted the manuscript. KR, AW, and RC contributed analyzing the data and critically revised the manuscript. All authors discussed the results and contributed to the final manuscript.

## Conflict of Interest

The authors declare that the research was conducted in the absence of any commercial or financial relationships that could be construed as a potential conflict of interest.

## Publisher's Note

All claims expressed in this article are solely those of the authors and do not necessarily represent those of their affiliated organizations, or those of the publisher, the editors and the reviewers. Any product that may be evaluated in this article, or claim that may be made by its manufacturer, is not guaranteed or endorsed by the publisher.
