# Supplementary material for: Neutrophil-to-Lymphocyte and Platelet-to-Lymphocyte Ratio in Univentricular Patients From Birth to Follow-Up After Fontan—Predicting Lymphatic Abnormalities
Source: Front Pediatr. 2021 Dec 8;9:740951. doi: 10.3389/fped.2021.740951 (PMC8692875; doi:10.3389/fped.2021.740951)
Supplement: Supplementary file 1 [file Table_1.pdf]

Supplementary Table 1: Laboratory measurements

	At Glenn surgery	At Cardiac catheterisation prior to Fontan	At Fontan surgery	At 6-month MRI
Haemoglobin	14.7±1.7	16.6±1.5	14.4±1.7	14.0±1.4
Uric acid	4.4±1.1	4.4±0.9	4.5±0.9	4.3±1.2
Total protein	60.7±5.4	66.2±4.7	65.8±5.2	64.9±8.2
Creatinine	0.3±0.1	0.3±0.1	0.4±0.1	0.4±0.1
Urea	19.5±7.4	26.6±7.1	27.3±8.1	27.0±8.1
Aspartate transaminase (AST)	39.2±8.8		36.2±6.2	
Alanine Aminotransferase (ALT)	23.3±10.8		17.4±8.9	
Gamma-glutamyl Transferase (GGT)	20.8±12.5		13.93±3.2	
